# From Anionic Ring-Opening Polymerization of β-Butyrolactone to Biodegradable Poly(hydroxyalkanoate)s: Our Contributions in This Field

**DOI:** 10.3390/polym13244365

**Published:** 2021-12-13

**Authors:** Grażyna Adamus, Adrian Domiński, Marek Kowalczuk, Piotr Kurcok, Iza Radecka

**Affiliations:** 1 Centre of Polymer and Carbon Materials, Polish Academy of Sciences, 34 M. Curie-Sklodowskiej Str., 41-800 Zabrze, Poland; grazyna.adamus@cmpw-pan.edu.pl (G.A.); adrian.dominski@cmpw-pan.edu.pl (A.D.); 2 Wolverhampton School of Science, Faculty of Science and Engineering, University of Wolverhampton, Wolverhampton WV1 1LY, UK; i.radecka@wlv.ac.uk

**Keywords:** β-butyrolactone, anionic ring-opening polymerization, poly(hydroxyalkanolate)s, biodegradable polymers

## Abstract

The feasibility of synthesis of functionalized poly(3-hydroxybutanoic acid) analogue and its copolymers via ring-opening polymerization of β-butyrolactone mediated by activated anionic initiators is presented. Using these new synthetic approaches, polyesters with a defined chemical structure of the end groups, as well as block, graft, and random copolymers, have been obtained and characterized by modern instrumental techniques, with special emphasis on ESI-MS. The relationship between the structure and properties of the prepared polymeric materials is also discussed.

## 1. Introduction

Over 35 years ago, some of us originally reported on the anionic ring-opening polymerization (ROP) of β-butyrolactone (BBL) into poly(β-butyrolactone) (poly(BBL)), which is an amorphous, atactic analogue of isotactic poly([R]-β-hydroxybutyrate) (PHB), a promising and widely used natural biodegradable polyester. It has always been worth noting that the anionic ROP of BBL is particularly difficult since the active centers are prone to deactivation. Over this period of time, our contribution to this field has been focused, among others, on: homo- and copolymerization of BBL with anionic initiators of different nucleophilicity; stereochemistry of anionic ROP of BBL to polymers of controlled microstructure; (bio)degradation of synthetic poly(BBL) and its blends; synthetic and natural poly(hydroxyalkanolate)s (PHA) as precursors of functionalized oligomers; as well as structural studies at the molecular level of synthetic and natural PHA by multistage mass spectrometry.

According to the different strategies focused on biodegradable PHA, we report herein the coherent approach divided into the above-mentioned sections. We believe that this review will provide a unique insight into the structural and compositional diversity of biodegradable PHAs, both synthetic and natural.

## 2. Our Contributions in This Field

### 2.1. Homo- and Copolymerization of BBL with Anionic Initiators of Different Nucleophilicity

**Scheme 1:**
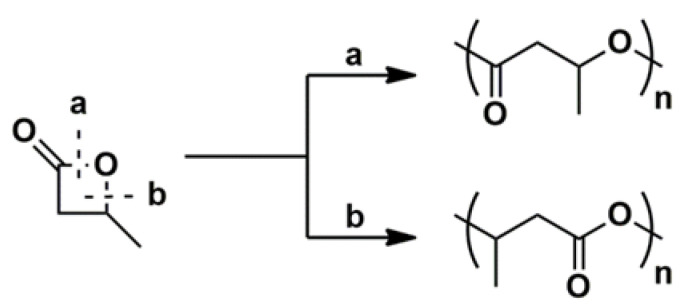
Two possible mechanisms for the ROP of BBL.

The ring opening of BBL can proceed by two different pathways: O-acyl cleavage (a) and O-alkyl cleavage (b), depicted in Scheme 1. From both pathways, it can be deduced that acyl cleavage leads to the formation of an alkoxide chain-end group, while a carboxylate results from alkyl cleavage.

**Scheme 2:**
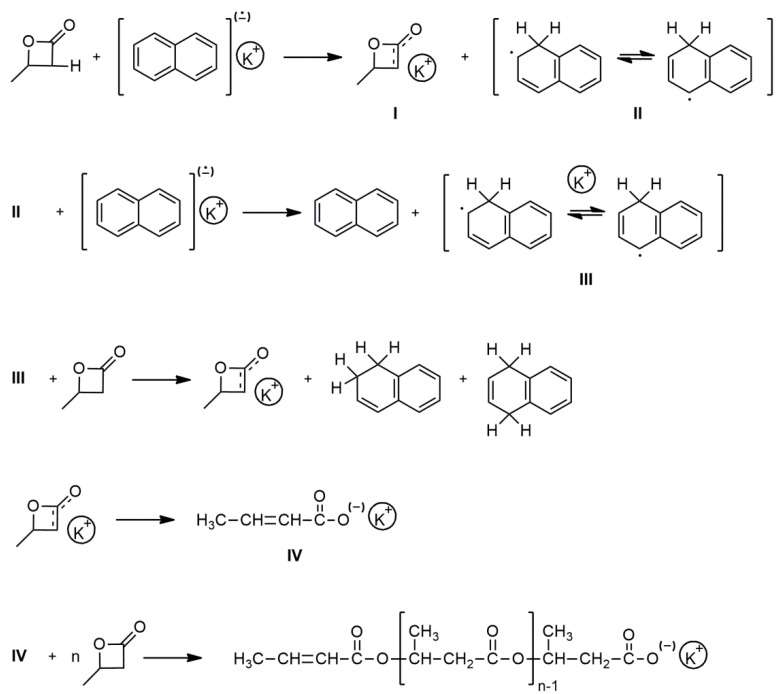
Anionic ROP of BBL in the presence of potassium naphthalenide/18-crown-6 complex.

Over 35 years ago, β-butyrolactone was polymerized by a homogeneous solution of potassium in THF. This solution results from the reaction of a crown ether (18-crown-6) in THF with a preformed potassium mirror [1,2]. The presence of a crown ether was found to be essential for the anionic ROP of this dormant monomer. Thus, alkali metal naphthalenides in THF, such as potassium naphthalenide complexes with crown ethers or cryptands, are another class of effective initiators for ROP of BBL. Accordingly, we prepared poly(BBL) within high yields and with a narrow molecular mass distribution. The mechanism of this polymerization consists of an α-proton abstraction from monomer, formation of the corresponding enolate, and subsequent scission of the alkyl-oxygen bond with formation of a potassium crotonate (Scheme 2). This species initiates propagation, which proceeds through an active carboxylate group [3,4].

A similar initiation mechanism was observed in anionic polymerization of BBL in the presence of a potassium hydride/18-crown-6 complex. The α-proton abstraction of the monomer was found to proceed at the initiation step of this polymerization. The salt of the crotonic acid formed initiates further propagation, leading to polyester functionalized with unsaturated dead end groups [5].

**Scheme 3:**
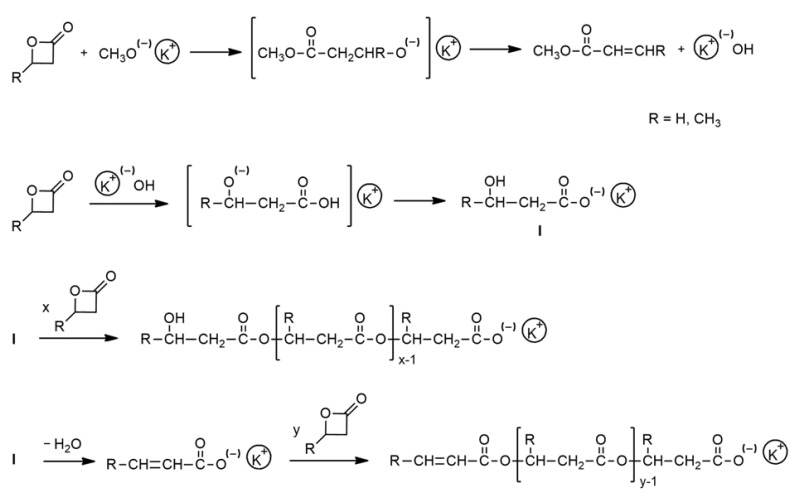
Anionic ROP of BBL in the presence of CH_3_-OK/18-crown-6 complex.

Over this time, it has also been shown that sodium and potassium alkoxides (strong nucleophiles) in aprotic solvents react with the BBL according to the mechanism of nucleophilic substitution at the carbonyl carbon with the acyl-oxygen bond of the lactone ring scission and formation of an unstable β-hydroxyester alkoxide, which is finally transformed into to the respective alkyl crotonate (Scheme 3). The alkali metal hydroxide formed in this reaction is the real polymerization initiator, and its reaction with the monomer molecule leads to the formation of a hydroxyacid salt. Under the reaction conditions, the salt is partially eliminated to the unsaturated acid salt. Thus, the chain growth in the studied polymerization occurs only at carboxylate centers [6,7,8,9]. 

The complexity of the initiation mechanism of anionic BBL polymerization, in relation to the ambient reactivity of the initiator used, BBL purity, and the effect of experimental parameters such as solvent and temperature, has been further studied in detail. The influence of monomer purity on the polymerization reaction reveals that an additional reaction of BBL with an oxidizing agent, such as potassium permanganate, produces a monomer of higher purity, as demonstrated by the higher rate of BBL polymerization and improved control over the polymerization process that is initiated with either tetrabutylammonium acetate or carboxylic acid/phosphazene base (P1-t-Bu, P2-t-Bu and P4-t-Bu) systems [10]. 

The anionic polymerization of BBL initiated with acetic acid salts in selected solvents showed a significant dependence of the activity of the initiator and polymer chain-growth centers on the size of the counterion used for the process carried out in a solvent with relatively low polarity (tetrahydrofuran). In addition, acceleration or retardation of the polymerization was found, depending on the initiator (counterion)/solvent system used. For a carboxylate with a small counterion in a solvent with high polarity and for a carboxylate with a large counterion in a solvent with low polarity, acceleration of the reaction was observed. However, for salts with a large counterion in a highly polar solvent, the opposite effect was observed [11]. Based on the information obtained, the method for the synthesis of high molar mass (Mn > 100,000) poly(BBL) with low dispersity by anionic ROP carried out in bulk and initiated with tetrabutylammonium acetate was described [12].

**Scheme 4:**
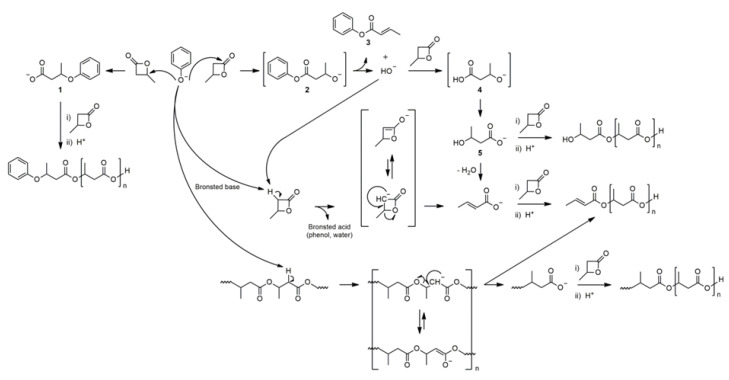
Anionic ROP of BBL initiated by sodium phenoxide (for simplicity, the counterion is omitted).

The mechanism of BBL polymerization initiated with both strong nucleophiles, e.g., alkali metal methoxide, and weak nucleophiles, such as alkali metal carboxylates, inspired us to clarify the mechanism of initiation of the polymerization of this monomer in the presence of solvent-activated (DMSO) initiators , such as alkali metal phenolates, i.e., weak nucleophiles/bases with an alkoxide structure of active centers [13]. The obtained results of the research on this system clearly show that the structure of the active centers of the anionic initiator does not affect the initiation mechanism, and the main factor defining the opening mechanism of the BBL ring is the basicity and nucleophilicity of the initiator (Scheme 4). 

The lowest basic phenoxide (sodium p-nitrophenoxide) reacts mainly as a weak nucleophile, i.e., the nucleophilic attack occurs at the C4 carbon of the lactone, similarly to weak-nucleophile-initiated polymerization. In the case of using sodium p-methoxyphenoxide, the most basic of the tested phenoxides (but also the most nucleophilic), the initiation proceeds predominantly according to the addition/elimination mechanism typical of strong nucleophiles, e.g., alkali metal alkoxides. It is important that depending on the basicity and nucleophilicity of the initiator used, the initiation of anionic polymerization of BBL varies, but ultimately, the centers of chain growth are carboxylate centers, which is important, for example, when planning the synthesis of block copolymers [13].

Our studies on anionic ROP of BBL have opened a new perspective for the preparation of bioactive polymer conjugates containing biodegradable polymer moieties. PHA and their synthetic analogues, due to their in vivo and in vitro biodegradation, as well as cell and tissue compatibility, can be used in medical applications, especially as drug delivery systems, implants, including heart-valve tissue engineering, vascular tissue engineering, bone tissue engineering, cartilage tissue engineering, as well as nerve conduit tissue biomaterials [14]. We have shown that poly(BBL) oligomers are non-toxic and can potentially serve to modify pharmacological properties and/or serve as carriers able to vectorize drugs in the form of chemical conjugates for drug delivery. The methods for oligomer preparation are simple, and drug sensitivity tests have proved that these conjugates are nontoxic. Moreover, poly(BBL) oligomers do not induce the cellular cytoprotective response [15,16]. 

Over 90 years after Fleming’s discovery, penicillins are still widely used as antibiotics, and a variety of their new derivatives have been synthesized, tested, and commercialized. This β-lactam antibiotic covalently bonded to atactic poly(BBL) was originally synthetized via ring-opening polymerization of racemic BBL initiated by a supramolecular complex of penicillin G potassium salt, thus showing that the conjugation of penicillins is a promising method of pharmacokinetic modification of such antibiotics [17,18]. 

The anticancer activity of acetylsalicylic acid with oligo p(BBL) conjugates, as well as their characteristics and in vitro biological studies, has also been reported [19]. Research has demonstrated that acetylsalicylic acid (aspirin) attached via hydrolysable ester bonds to non-toxic, well-defined BBL oligomers was more effective than aspirin in growth inhibition of human colon adenocarcinoma cells, HT-29, and human colon carcinoma cells, HCT 116, in vitro. 

Ibuprofen is a well-known anti-inflammatory, analgesic, and antipyretic drug that has recently been found to slow down proliferation of colon cancer cells effectively. A convenient method of synthesis of ibuprofen conjugates via anionic oligomerization of BBL initiated with ibuprofen sodium salt in DMSO, as well as their antiproliferative activity against HT-29 and HCT 116 colon cancer cells, has been studied. Research has demonstrated that such modification increased the anticancer potential of the drug. A significant difference between the antiproliferative properties of ibuprofen conjugate, as compared to free ibuprofen, indicates changes in the mechanism of action or bioavailability of the drug caused by the attachment of 3-hydroxybutyrate oligomers, as well as enhancement of cellular uptake of ibuprofen conjugate [20]. 

The above-mentioned examples demonstrate that conjugation of poly(BBL) oligomers with selected drugs could be one strategy aimed at improving or modifying biopharmaceutical properties of the drug.

### 2.2. Studies on the Biodegradable Polymer Systems for Controlled Release of Bioactive Substances for Cosmetology

Using the concept of activated initiators in anionic polymerization of BBL and extension on other β-substituted β-lactones containing a bounded biologically active substance, innovative, biodegradable polymer systems were developed for controlled release of bioactive substances for application in cosmetology. Two approaches were elaborated to enable chemical bonding of the biologically active substances of antioxidative properties with the chains of oligomeric 3-hydroxybutyrate and its copolymers. In the first method, as initiators of the ring-opening polymerization of β-butyrolactone, the sodium or potassium salts of the selected phenolic acids with antioxidative properties were used.

Conjugates were obtained in which the biologically active compounds of antioxidative properties, including lipoic acid, as well as selected phenolic acids, were chemically bonded as end groups of the oligomeric poly(BBL) [21,22]. In further research, within the cooperation of the team managed by Professor Janusz Jurczak from the Institute of Organic Chemistry of the Polish Academy of Sciences, a method for synthesis of β-substituted β-lactones containing biologically active substances was developed, which has not yet been described in literature [23]. Using the β-substituted β-lactones containing a chemically bonded bioactive substance as monomers or comonomers in anionic polymerization obtained bioactive (co)oligoesters containing more molecules of the bioactive substance linked as side groups along the polymer chain [24]. It was demonstrated that the studied conjugates are non-toxic and well-tolerated by epidermal cells. The conducted tests of permeability confirmed that the obtained bioconjugates penetrated deep into skin layers, but no in vitro transdermal penetration was observed [22].

### 2.3. Studies on the Biodegradable Polymer Systems for Controlled Release of Bioactive Substances for Agriculture and Environmental Protection

Simultaneous to the research on the systems for controlled release of bioactive substances for cosmetology, studies on systems for controlled release of bioactive substances for potential application in agriculture were conducted. Research covered bioactive substances belonging to pesticide groups, which aimed to develop systems to allow for extended duration of release of a pesticide for the purpose of delivering its optimum amount to a target area. As a result, this would limit significant losses of active ingredients caused by weather conditions, as well as the adverse impact of such compounds on the environment, which can be seen in conventional forms of pesticide use. Preliminary research resulted in the development of methods that allow us to chemically bind the bioactive substances, i.e., pesticides with biodegradable oligomers of poly(BBL)s. The developed methods are based on anionic oligomerization of BBL or other β-substituted β-lactones containing linked bioactive substance selected from the pesticide group and possessing a carboxyl group. Just like in the case of the above-mentioned conjugates for cosmetology, the application of these methods allowed us to obtain and characterize two types of pesticide-oligomer conjugates:

(i) pesticide-oligo BBL conjugates in which each oligo(BBL) chain contains one molecule of pesticide (a herbicide or an antibacterial substance) as an end group, which is connected by ester bond [25,26],
(ii) homo- and (co)oligoesters containing an increased amount of bioactive substance connected to the oligomer chains as the end group and side groups along the polymer chain. The amount of pesticide molecules bonded to the chain of (co)oligoesters may be controlled by changing the composition of the β-lactone monomers used in the process of (co)oligomerization [27].

Initial tests on the obtained conjugates were performed to determine the usefulness of the developed systems for application in agriculture. An important element was research into hydrolytic degradation of pesticide-oligomer conjugates, which confirmed that the ester bond between the bioactive substance and the oligomer chain is subject to hydrolysis. This allowed for the gradual release of the bioactive compounds from the oligomer chain in their original form, while maintaining their biological activity. Moreover, the tests conducted in both greenhouse and field conditions, in cooperation with the Plant Protection Institute in Sośnicowice and the Jan Długosz University in Częstochowa, Poland, demonstrated that the herbicidal effectiveness of the formulations containing the selected polymer-herbicide conjugates on selected dicotyledonous weeds was comparable to that of the commercially available formulation containing the same active substance [28].

The benefit of the developed system is an extended period of action, which permits a reduction in the use of that type of ingredients during one season.

The positive results of these preliminary tests of the oligomer-pesticide conjugates obtained through anionic oligomerization provided the impulse for continuing research in this area and to search for synthesis methods of such conjugates that might be more economically advantageous. This resulted in the development of a method for synthesis of this type of conjugates through transesterification of commercially available aliphatic biopolyesters with selected pesticides. The developed method is relatively simple, provides a high percentage value of attachment of pesticide to PHA, and may be applied to the synthesis of conjugates both using pesticides containing a carboxyl group and bioactive substances containing hydroxyl groups [29,30].

Such methods are promising both from an economical point of view and from the point of view of scale-up of synthesis, owing to the application of commercially available polyhydroxyalkanoates and pesticides used in preparations currently available on the market, the relatively short period of reaction of biopolyesters with pesticides (transesterification in 2 min), and the elimination of solvents. 

### 2.4. Varying Copolymer Composition Affords Copolyesters with Adjustable Properties

The block polymerization of BBL with β-propiolactone proceeds fast, with a high yield in the presence of potassium solutions in THF containing 18-crown-6. Respective block copolymers with the expected molar mass and composition are formed in this way. Their glass transition and melting temperatures, as well as their melting enthalpies, determined by DSC, show a strict correlation with block copolymer composition [31]. 

The “living” poly(BBL) with carboxylate active centers was applied as an initiator for synthesis of poly(pivalolactone) (PPVL) block copolymers. The obtained diblock copolymers with tailored molecular weight and composition contain an amorphous phase with Tg = 5 °C, associated with the poly(BBL) block, and a high-melting crystalline phase, the amount of which increases with PPVL content [32].

Copolyesters with designed architecture were obtained via anionic ring-opening copolymerization of BBL with β-ethoxymethyl-β-propiolactone initiated with tetrabutylammonium acetate. Depending on the reaction conditions, diblock or random copolymers were obtained, and their structure was evaluated at the molecular level [33]. 

**Scheme 5:**
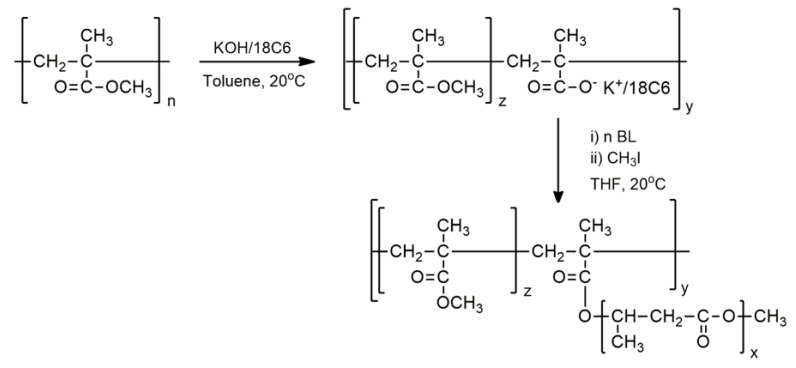
Synthesis of graft copolymers of PMMA with poly(BBL) side chains.

Diblock copolymers consisting of natural PHAs and poly(BBL) were also prepared. Macroinitiators obtained by the controlled degradation of natural PHAs (PHB, PHBV, or PHO) in the presence of KOH/18-crown-6 complex were then used in anionic ROP of BBL [34]. Combining the anionic polymerization of the of BBL with the coordination ring-opening polymerization of the *ε*-caprolactone enabled synthesis of the respective diblock copolymer. According to NMR and DSC analysis, the poly(BBL) block is atactic and totally amorphous, in contrast to the polycaprolactone block, which is semicrystalline. However, a partial miscibility of the two blocks in the amorphous phase was detected [35]. The polyethylene oxide (PEG)-containing poly(BBL)-b-PEG-b-poly(BBL) triblock copolymers were obtained via anionic polymerization of racemic BBL, initiated with respective PEG macroinitiators with carboxylate moieties. The structure of resulting triblock copolymers was proved by SEC and NMR spectroscopy [36]. Star-like copolymers composed of hydrophilic PEG and hydrophobic poly(BBL) segments linked by phosphoester moiety were also obtained [37]. A brush copolymer composed of a biodegradable hydrophobic poly(BBL) chain and hydrophilic PEG brushes was synthesized by a three-step procedure consisting of ring-opening anionic polymerization of BBL, yielding polyester with two hydroxyl functionalities at one chain terminus, followed by synthesis of PHB-derived microinitiator species, and ATRP of polyethylene glycol methyl ether methacrylate. The self-aggregation behavior of the brush copolymer in an aqueous medium was evidenced by optical-absorption-probe technique and dynamic/static light-scattering measurements [38]. ATRP was also used for synthesis of the poly(BBL) copolymers modified by introduction of hydrophobic or hydrophilic segments via grafting technique [39]. The anionic grafting from the reaction of BBL on poly(methyl methacrylate) (PMMA) was used for the synthesis of graft copolymers of PMMA with poly(BBL) side chains [40]. The partially saponified PMMA bearing carboxylate anions complexed by 18-crown-6 potassium counterion was used as a macroinitiator of BBL polymerization (Scheme 5). The compatibilizing effect of such graft copolymers on bacterial PHB/PMMA blends was observed [41]. 

New electroactive polymeric materials containing BBL oligomers were synthesized for potential biomedical applications. The discussed materials describe BBL oligomers grafted to the polymer matrix consisting of conductive (co)polypyrrole. The influence of grafting density and molar mass of oligo(BBL) brushes on the physicochemical properties, e.g., electrical conductivity and degradation of prepared copolymers, was also investigated [42,43]. 

### 2.5. Stereochemistry of Anionic ROP of BBL to Poly(BBL) of Controlled Microstructure

**Figure 1:**
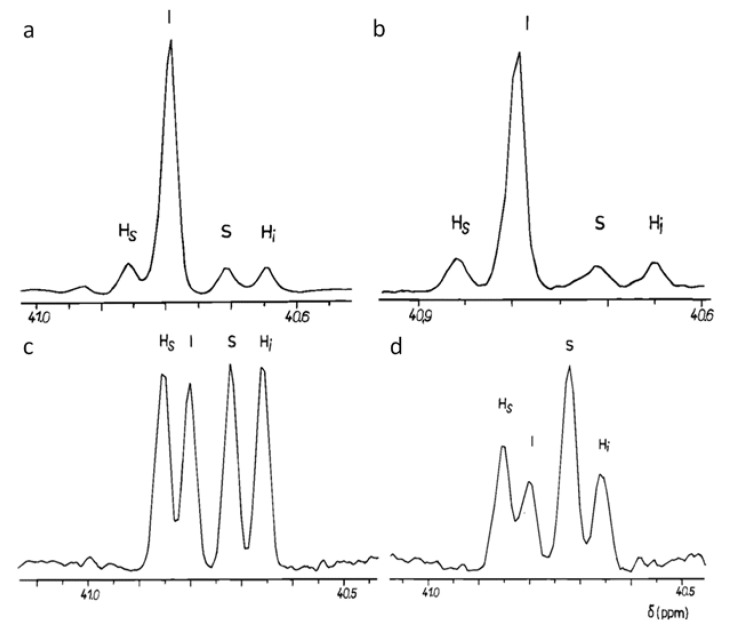
^13^C NMR spectra (methylene carbon region) of poly(BBL)s obtained via polymerization of (R)-BBL initiated with 18-crown-6 supramolecular complexes of (**a**) potassium methoxide and (**b**) potassium acetate; and poly(BBL)s obtained by polymerization of (R,S)-BBL initiated with 18-crown-6 supramolecular complex of potassium methoxide (**c**) at 20 °C, (**d**) at −10 °C (methanol-insoluble fraction). (Modified with permission from Ref. [45]. Copyright 1996, American Chemical Society).

PHAs are produced in bacteria and mammalian organisms, and these polymers play an important role in many biochemical processes. It is believed that PHB in living cell membranes forms channels responsible for transportation of metal ions [44]. As mentioned above, natural isotactic PHB contains repeating units with only *R* configuration at the *β* position, while amorphous atactic poly(BBL) formed via anionic ROP of racemic BBL contains randomly distributed (R) and (S) units. Isotactic poly(S-BBL) was prepared chemically by anionic ROP of optically active (R) BBL (90% *R* + 10% *S*). Predominantly syndiotactic poly(BBL), the polymer containing alternating sequences of (*R*)- and (*S*) *β*-hydroxybutyrate-repeating units, was also synthesized by way of anionic ROP with equimolar amounts of tartarate esters with respect to the initiator or in the reaction carried out at −10 °C [45]. The microstructure of the obtained polymers was established by ^13^C NMR (Figure 1). As a consequence of the above findings, the first facile synthesis of biomimetic poly([R]-BBL) via regioselective anionic polymerization of [S]-BBL was reported. Using [R]-3-hydroxybutyric acid sodium salt complex with 18-Crown-6 as initiator, the analogue of natural PHB, with the same chemical structure of the end groups and isotactic poly-[R] microstructure, was prepared [46]. The low-molar-mass poly([R]-BBL) was used for obtained lipid bilayers formation [47].

### 2.6. (Bio)degradation of Synthetic Poly(BBL) and Its Blends

The abiotic hydrolytic degradation properties of synthetic poly(BBL), atactic and predominantly syndiotactic, in comparison with natural PHB, were evaluated. It was found that the chemical microstructure has no substantial influence on the mechanism of hydrolytic degradation. Regardless of the microstructure of the polymers studied, the hydrolytic degradation takes place via random scission of the polyester chain. However, the degree of polymer crystallinity influences the rate of hydrolytic degradation [48]. 

Atactic poly(BBL) was found to be a valuable component of blends with other biodegradable polymers. Its blends with a natural bacterial PHBV were miscible in the melt and solidified with spherulitic morphology. The influence of poly(BBL) content on the thermal and mechanical properties of the blends was evaluated. Enzymatic degradation experiments revealed that the degradation rate of the blends was higher than that of PHBV and increased with poly(BBL) content in the blends, whereas poly(BBL) did not biodegrade under these conditions [49]. However, water-soluble BBL oligomers were found to be bio-assimilated by bacterial strains of not only two PHB-degrading bacteria (*Alcaligenes faecalis T1* and *Comamonas* sp.) but also a non-PHB-degrading bacterium (*Ralstonia eutropha H16*) [50]. Further studies revealed that a novel type of hydrolase (*phaZ7*) expressed an unusual substrate specificity to atactic poly(BBL) oligomers [51]. 

### 2.7. Natural PHAs as Precursors of Functionalized Oligomers

**Scheme 6:**
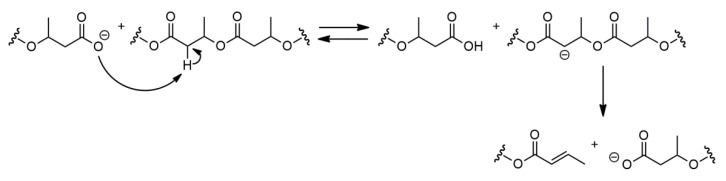
E1cB mechanism of PHB degradation.

A novel intermolecular degradation reaction of poly(BBL) and its homologs was discovered and described, and its mechanism was proposed. The degradation reaction, which takes place at moderate temperatures, follows the intermolecular E1cB mechanism and is “initiated” by factors capable of abstracting a proton at the C2 carbon of the polyester chain (Scheme 6) [52,53].

Investigations of the kinetics of PHB degradation induced by carboxylates showed that due to the low activation energy, this reaction can proceed at relatively low temperatures. The conducted research also allowed for the determination of the dependence of the apparent activation energy of the degradation process on the size of the counterion in the carboxylate [54]. Moderate-temperature degradation studies have demonstrated the utility of Bronsted-Lowry weak acid salts for the controlled production of PHB macromonomers with a crotonate end group. For fast degradation under mild conditions, the most effective compounds turned out to be alkali metal salts. Moreover, an efficient method of controlled production of PHA oligomers with controlled molar masses in a continuous process using an extruder was developed. Such oligomers are potentially valuable precursors (macromonomers) for the synthesis of new polymeric materials for medical applications [55]. 

Crotonate-terminal-functionalized oligomers produced as a result of degradation of PHB occurring at moderate temperatures (according to the E1cB mechanism) are of little use due to the relatively low reactivity of the crotonate moiety. In order to transform this group into a functional group of much higher reactivity, studies of its selective oxidation (in the obtained oligomers) were performed. An effective method was developed to obtain poly(3-hydroxybutyrate) functionalized with reactive 3-methyloxirane-2-carboxylate end groups as a result of oxidation of PHB crotonate with m-chloroperbenzoic acid [56]. 

Investigations of PHB crotonate ozonolysis, combined with the decomposition of its peroxidic products, have shown that it is a fast, effective, selective, and quantitative method of obtaining PHB functionalized with glyoxylate and mono oxalate end groups, respectively, depending on the method of decomposition reactions of peroxides generated in ozonolysis [57]. The reactions of the obtained PHB derivatives with model amines and alcohols showed that they can be used in the synthesis of conjugates of bioactive substances or in the modification of polymers containing groups capable of reacting with the functional groups of the obtained macromonomers, e.g., –OH or –NH_2_. Moreover, it was found that the imine group formed as a result of the reaction of the oligomer glyoxylate group with the primary amine can be used in pH-controlled drug delivery [57].

The study of PHB degradation in a mixture of ozone and oxygen at elevated temperatures showed that it leads to a mixture of oligomeric products. Originally, the presence of malic-acid units incorporated into the polymer main chain, formed as a result of the oxidation of the methyl group of the 3-hydroxybutyrate constitutional units, was found in the reaction products. Therefore, it was shown that this degradation process is an interesting method of obtaining poly(3-hydroxybutyrate-co-malic acid) [58].

New biodegradable copolymers for the production of pH-sensitive drug delivery systems using PHB oligomers were designed and synthesized. It is well known that due to the Warburg effect in cancer cells, the tumor and peritumoral environment is characterized by a slightly lower pH than that of healthy cells. Therefore, the concentration of hydrogen ions should trigger the release of the drug in the developed systems. The poly(ethylene glycol)-b-polycarbonate-b-oligo([R]-3-hydroxybutyrate) was synthesized by PEG/organocatalyst initiated ring-opening polymerization of ketal protected six-membered cyclic carbonate (PC) followed by esterification with oligo([R]-3-hydroxybutyrate). The amphiphilic PEG-PC-PHB copolymer self-assembles into micelles with a diameter of approximately 25 nm. Acid-catalyzed hydrolysis of the acetal moiety in the carbonate block increases the hydrophilicity of the hydrophobic part of the micelles, leading to swelling of the micelles and drug release. It was shown that the introduction of the PHB block significantly increases the stability of micelles in the entire range of the tested pH. The drug-release profiles in buffers of different pH levels showed a significant dependence of the hydrolysis rate of the tested materials on the pH of the environment. It was found that unloaded micelles are non-toxic, while micelles with an active substance (doxorubicin or 8-hydroxyquinoline glycoconjugates) show a significant increase in inhibition of the proliferation of MCF-7 and HCT-116 tumor cells compared to the pure drug [59].

An original method for selective reduction of PHA biopolyesters, leading to attainment of uniform, linear, telechelic PHA oligomers, ended with two hydroxyl groups—oligo estrodiols with designated chemical structure and controlled molar mass [60,61]. This method constitutes a source of PHB oligodiols useful in the synthesis of new tailor-made biomaterials and may be also used for modification of the PHA surface by generating free hydroxyl groups on the outermost face. The presence of hydroxyl groups on the polymer surface was confirmed by ATR-FTIR studies, as well as contact-angle measurement. As expected, the change in surface chemistry improved the hydrophilicity of the polymer surface.

### 2.8. Synthesis of Natural PHA from Polyolefin Wastes

Applications of petroleum-based plastics comes with severe environmental consequences due to their recalcitrance to biodegradation, which leads to their accumulation in different environmental compartments (terrestrial and aquatic) in high quantities and difficulties in managing them. We recently systematically exploited inexpensive carbon sources, especially waste materials, for sustainable and cheap production of PHAs [62]. Among the waste products currently being exploited is post-consumer plastic waste. Research has been reported on the utilization of post-consumer plastic waste, such as oxidized polyethylene wax [63], low-density polyethylene wax [64], as well as oxidatively fragmented polystyrene [65], polypropylene [66], and polyethylene [67], for the production of PHAs. ESI-MS was also used for structural characterization of the obtained PHA. NMR and ESI-MS/MS analyses revealed that bioconversion, as guided by *Cupriavidus necator*, led to the production of PHA-random copolymers. The reported findings give rise to an interesting breakthrough in the production of valuable biodegradable/compostable polymeric materials, such as PHAs and relevant plastic items.

### 2.9. Mass Spectrometry Molecular Studies of PHA and Their Synthetic Analogues

Among modern instrumental techniques, soft ionization mass spectrometry represents a powerful toolset that is particularly suited for the structural characterization of polymers described in this review.

The application of mass spectrometry with “soft ionization techniques” for structural studies of aliphatic biopolyesters and their chemical analogues led to the development of methods allowing for the determination of the structure of these polyesters at the molecular level. One of the most important achievements in the field of characterization of aliphatic copolyesters has been the development of a method for determining the structure of a selected aliphatic PHA biopolyester at the molecular level by means of mass spectrometry. Research included biopolyesters of PHBV (3-hydroxybutyric acid with 3-hydroxyvaleric acid), PHO (3-hydroxyoctanoic acid with 3-hydroxyoctanoic acid), and PHBH (3-hydroxybutyric acid with 3-hydroxyhexanoic acid). 

In structural studies, the oligomers of biopolyesters PHBV, PHBH, and PHO formed as a result of controlled degradation were used. [52,68]. Analysis of the mass spectra obtained for these oligomers provided important information on the structure of individual biopolyester chains, which allowed for the verification of the chemical homogeneity and chemical composition of the tested biopolyesters [68,69,70,71]. The chemical composition and sequence distribution of comonomeric units in the studied biopolyesters were determined on the basis of a comparison of the experimental mass spectra obtained for the tested biopolyesters by means of ESI-MS and the spectra generated with the assumption of Bernoulli statistics [69,72]. An original solution proposed as a result of the structural research of PHA biopolyesters was the use of the electrospray ionization multi-stage mass spectrometry (ESI-MS^n^) technique. Fragmentation of selected molecular ions of the PHA biopolyester (using the technique of multi-stage mass spectrometry) made it possible to confirm the random distribution of comonomeric units in the studied biopolyesters [68,69,70,72]. The results of the structural studies carried out with the use of MS techniques and their correlation with the results obtained by the ^1^H and ^13^C NMR methods led to the development of a protocol for studying the structure of PHA biopolyesters. Currently, the elaborated method is used in the structural study of new biotechnologically synthesized PHA biopolyesters, including those obtained with the use of renewable raw materials [65,66,73]. 

The application of ESI mass spectrometry in structural studies of the products obtained as a result of anionic polymerization of BBL using typical activated anionic initiators allowed for the determination of the structure of individual chains of the obtained poly(BBL). Identification and determination of the chemical structure of end groups allowed for unequivocal determination of the homogeneity of the obtained polyesters, resulting from the initiator applied in polymerization. For verification of the structure of the end groups, fragmentation of selected poly(BBL) ions, both with hydroxyl and crotonate end groups were performed in a mass spectrometer [74,75]. The precise determination of the chemical structure of end groups of poly(BBL) played a significant role in verification of the mechanism of the anionic polymerization of BBL [76,77]. In further research, mass spectrometry was applied to the study of the chemical structure of individual macromolecules of the polyesters obtained as a result of anionic polymerization of α,β-alkyl-substituted and β-alkoxy-substituted β-lactones, which significantly contributed to generalization of the mechanism of anionic polymerization of BBL, as well as to those lactones [78,79,80,81]. 

Structural studies conducted with the aid of mass spectrometry confirmed that regardless of the anionic initiator used, the propagation stage in the anionic polymerization of β-butyrolactone takes place on carboxylate centers. This finding initiated further work concerning the synthesis of poly(BBL) with assumed chemical structures of end groups, including bioactive end groups [42,82]. Application of the concept of activated initiators in anionic polymerization of BBL and β-substituted β-lactones containing a bonded, biologically active substance contributed to the development of innovative biodegradable polymer systems for controlled release of bioactive substances for application in medicine [17,20,82], cosmetology [21,22,24,83,84], and agriculture [25,26,27,29]. Structural studies of the obtained bioactive polymers by means of mass spectrometry allowed for the verification of their structure at the molecular level, which provides new research possibilities concerning the fundamental importance of macromolecular chemistry, as well as useful information of significant importance to future applications [22,24,84]. 

ESI-MS^n^ was also applied to the structural study of synthetic aliphatic copolyesters (such as copolymers of BBL with β-substituted β-lactones or L-lactic acid) with different chain architecture (blocky or random copolymers) [33,85,86]. The aim of these studies was to find a correlation between the structure of copolyester and the fragmentation pathways of of individual copolyester macromolecules.

**Figure 2:**
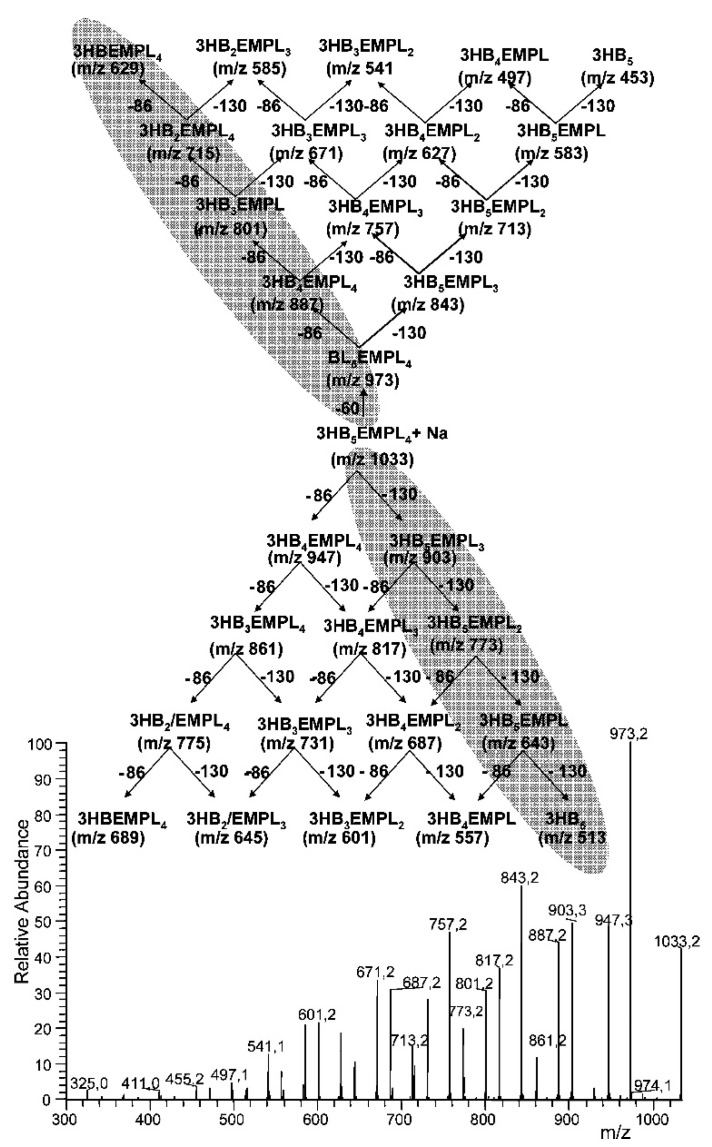
ESI-MS/MS spectrum of the sodiated precursor ion [3-HB_5_/EMPL_4_ + Na]^+^, m/z 1033, selected from the simple ESI mass spectrum (series A) of a random copolyester sample. Theoretical fragmentation pathway of the selected ion. The part of the scheme indicated in gray shows the product ions formed from BBL-β-ethoxymethyl-β-propiolactone diblock copolyester molecular ions at m/z 1033. (Reprinted with permission from Ref. [33]. Copyright 2009, American Chemical Society).

It was demonstrated that the arrangement of comonomer units along the copolyester chains was reflected in the profile of products formed as a result of the fragmentation of individual copolyester molecule ions in a mass spectrometer (Figure 2) [33,81]. The result of the conducted studies was the development of a general method for determining the sequence distribution of comonomer units in aliphatic copolyesters with different chain architecture (from diblock to random) [87]. Mass spectrometry was found also to be an effective tool for determining the structure of new graft copolymers made up of poly(γ-glutamic acid) as the main chain, to which oligohydroxybutyric side chains or bioactive substances were grafted, dedicated for application as biomaterials for development of systems for controlled release of biologically active substances [30,88]. 

The systematic development of research on the synthesis of aliphatic polyesters for medical applications observed in recent years has led to the development of a wide range of new biodegradable materials with the desired architecture and controlled properties. However, both the type and amount of products released during hydrolysis may affect the biocompatibility of such materials in their applications. Therefore, from the point of view of the selection of materials for specific applications, especially as bioresorbable materials, it is very important to precisely define the structure of products formed as a result of hydrolytic degradation.

An important element of the studies conducted in this scope is precise determination of the structure of the products generated as a result of degradation of polymeric materials containing PHA biopolyesters and their synthetic analogues. The application of mass spectrometry techniques with “soft” ionization methods in structural studies of degradation products allowed for the detection of even small amounts of degradation products released from biodegradable materials. It was demonstrated that the products of heterogenous enzymatic hydrolysis of synthetic poly(BBL) were the water-soluble higher oligomers of 3-hydroxybutyrate (up to heptamer), containing hydroxyl and carboxylate end groups [49]. Moreover, the conducted research demonstrated that model poly(BBL) oligomers are subject to biological assimilation by selected strains of bacteria [50,51].

In addition, mass spectrometry was also used to study thermal degradation of PHA biopolyesters. MS studies of the obtained degradation products showed that in the presence of carboxylic salts, the process of thermal degradation of PHA biopolyesters follows the α-deprotonation mechanism and leads to linear PHA oligomers with crotonate and carboxylate end groups. [52,53]. Within the studies conducted on controlled degradation of PHA biopolymers, the original method for selective reduction of PHA biopolyesters was developed. MS studies confirmed that reduction of PHA biopolymers with the aid of lithium borohydride led to uniform, linear, telechelic PHA oligo estrodiols with designated chemical structure and controlled molar mass [60,61]. 

The application of mass spectrometry in the study of degradation products of PHA biopolyesters, their chemical analogues, and polymeric materials containing structural fragments derived from atactic poly([R,S]-3-hydroxybutyrate) and PHA biopolyesters allowed for the establishment of the relationship between the structure of the developed polymer materials and their behavior under (bio)degradation processes.

## 3. Conclusions

The research that was the subject of this review was conducted over the last 30 years under the guidance of or cooperation with globally recognized scientists. Some of them have already passed away, and we would like to mention, in particular, Professor Zbigniew Jedliński from our center (1922–2008), Polish-born Professor Michael Szwarc from the University of Southern California, USA (1909–2000), Professor Robert W. Lenz from the University of Massachusetts at Amherst, USA (1926–2010), and Professor Emo Chiellini from the University of Pisa, Italy (1937–2020). We sincerely believe that this exciting area of knowledge has become more and more important for the current circular economy and will be further developed by our younger colleagues in the years to come [89,90]. 

## 4. Patents

1. Kwiecień, I.; Kowalczuk, M.; Adamus, G.; Kowalski, W.J.; Rychter, P.; Siłowiecki, A. Biologically active substance with herbicidal activity and the method of its manufacturing. PL 236741 B1. Date of filing: 18 July 2013. Date of publication: 8 February 2021 WUP 03/21.
2. Kowalczuk, M.; Adamus, G.; Kwiecień, I.; Maksymiak, M.; Jurczak, J.; Bałakier, T. New biodegradable (co)polyesters from beta-lactone monomers containing biologically active substances. PL 225906 B1. Date of filing: 15 September 2014. Date of publication: 30 June 2017 WUP 06/17.
3. Michalak, M.; Marek, A.A.; Zawadiak, J.; Kurcok, P.; Kowalczuk, M.M. Method for the preparation of poly(3-hydroxyalkanoate) macromonomers. PL 224368 B1. Date of filing: 8 November 2011. Date of publication: 30 December 2016 WUP 12/16.
4. Michalak, M.; Kurcok, P.; Kowalczuk, M.; Marek, A.A.; Zawadia, J. Functionalized polyhydroxyalkanoate macromonomers, method of their preparation and their application. PL 221 159 B1. Date of filing: 22 June 2012. Date of publication: 29 February 2016 WUP 02/16.
5. Kowalczuk, M.; Adamus, G.; Maksymiak, M.; Zawadiak, J.; Kwiecień, M.; Kurcok, P. Oligomer conjugates, preferably those for providing the skin with protective substances and the method of their manufacturing. PL 219 683 B1. Date of filing: 9 February 2011. Date of publication: 30 June 2015 WUP 06/15.
6. Kowalczuk, M.; Kurcok, P.; Adamus, G.; Kawalec, M.; Scandola, M.; Focarete, L.; Mazzocchetti, L. Process for controlled degradation of polyhydroxyalkanoates and products obtainable therefrom. EP 2 346 922 B1. Date of filing: 15 October 2008. Date of publication: 5 March 2014 Bulletin 2014/10.
7. Kurcok, P.; Kowalczuk, M.; Kawalec, M.; Sobota, M.; Michalak, M. Method for purifying β-butyrolactone, especially for the synthesis of poly(3-hydroxybutyrate) and its copolymers. PL 216 552 B1. Date of filing: 27 January 2011. Date of publication: 30 April 2014 WUP 04/14.
8. Kowalczuk, M.; Kurcok, P.; Adamus, G.; Kawalec, M.;Scandola, M.; Focarete, L.; Foltran, I. Method of controlling thermal degradation of anionically terminated polymers and materials obtained thereof. EP 1 999 189 B1. Date of filing: 20 March 2006. Date of publication: 11 November 2009 Bulletin 2009/46.
9. Jedliński, Z.; Łuczyk-Juzwa, M.; Zawidlak-Węgrzyńska, B.; Kaczmarczyk B.; Bosek, I.; Wanic, A. Esters of non-steroid anti-inflammatory medicines and the methods of their preparation. EP 1679301 A1. Date of filing 30 August 2005. Date of publication: 12 July 2006 Bulletin 2006/28.
10. Jedliński, Z.; Kurcok, P.; Kowalczuk, M. Method of obtaining amorphous poly([R,S]-3-hydroxy butyric acid). PL199104 B1. Date of filing: 19 April 2000. Date of publication: 29 August 2008 WUP 08/08.
11. Jedliński, Z.; Kurcok, P. Method of obtaining poly([R]-3-hydroxy butyric acid), an analog of a natural polymer. Date of filing: 28 January 1998. Date of publication: 30 June 2005 WUP 06/05.
12. Method of obtaining syndiotactic polybutyrolactone. PL 172 412 B1. Date of filing: 6 December 1993. Date of publication: 30 September 1997 WUP 09/97.
13. Jedliński, Z.; Kurcok, P.; Kowalczuk, M. Method for the production of polyester macromonomers. PL 160092 B1. Date of filing: 15 May 1989. Date of publication: 26 February 1993 WUP 02/93.
14. Jedliński, Z.; Kurcok, P.; Kowalczuk, M. Method of polyesters producing. PL 140 289. Date of filing: 12 April 1984. Date of publication: 30 February 1988.

## Data Availability

Data sharing not applicable.
